# EBV-LMP1 targeted DNAzyme enhances radiosensitivity by inhibiting tumor angiogenesis via the JNKs/HIF-1 pathway in nasopharyngeal carcinoma

**DOI:** 10.18632/oncotarget.3331

**Published:** 2015-01-21

**Authors:** Lifang Yang, Liyu Liu, Zhijie Xu, Weihua Liao, Deyun Feng, Xin Dong, San Xu, Lanbo Xiao, Jingchen Lu, Xiangjian Luo, Min Tang, Ann M. Bode, Zigang Dong, Lunquan Sun, Ya Cao

**Affiliations:** ^1^ Cancer Research Institute, Key Laboratory of Chinese Ministry of Education, Xiangya School of Medicine, Central South University, Changsha, China; ^2^ Center for Molecular Medicine, Xiangya Hospital, Central South University, Changsha, China; ^3^ Department of Radiology, Xiangya Hospital, Central South University, Changsha, China; ^4^ Department of Pathology, Xiangya Hospital, Central South University, Changsha, China; ^5^ The Hormel Institute, University of Minnesota, Austin, MN, USA

**Keywords:** LMP1, DNAzyme, radiosensitivity, JNK, angiogenesis, NPC

## Abstract

LMP1, which is encoded by the Epstein-Barr virus, is proposed to be one of the major oncogenic factors involved in nasopharyngeal carcinoma (NPC). Previous studies demonstrated that down-regulation of LMP1 by LMP1-targeted DNAzyme (DZ1) increases the radiosensitivity of NPC. However, the mechanism by which DZ1 contributes to this radiosensitivity remains unclear. In this study, we determined whether a DZ1 blockade of LMP1 expression has an overall positive effect on the radiotherapy of NPCs by repressing HIF-1/VEGF activity and to investigate the mechanisms underlying LMP1-induced HIF-1 activation in NPC cells. The results showed that DZ1 inhibited the microtubule-forming ability of HUVECs co-cultured with NPC cells, which occurs with the down-regulation of VEGF expression and secretion. Moreover, LMP1 increases phosphorylated JNKs/c-Jun signaling, which is involved in the regulation of HIF-1/VEGF activity. After silencing LMP1 and decreasing phosphorylation of JNKs, NPC cells exhibited an enhanced radiosensitivity. Furthermore, *in vivo* experiments revealed a significant inhibition of tumor growth and a marked reduction of the K^trans^ parameter, which reflects the condition of tumor micro-vascular permeability. Taken together, our data suggested that VEGF expression is increased by LMP1 through the JNKs/c-Jun signaling pathway and indicated that DZ1 enhances the radiosensitivity of NPC cells by inhibiting HIF-1/VEGF activity.

## INTRODUCTION

Nasopharyngeal carcinoma (NPC) is a malignancy most strongly associated with the Epstein-Barr virus (EBV), and radiotherapy is the most effective treatment against NPC. However, due to radioresistance, loco-regional recurrence or metastatic spread still occurs in a high proportion of patients who undergo radiotherapy [1]. Latent membrane protein 1 (LMP1) is an EBV-encoded protein with oncogenic properties [2]. LMP1 plays an essential role in tumorigenesis of NPCs through the activity of various signal pathways and is thus a potential target for NPC biotherapy [3-5]. Down-regulation of LMP1 was shown to be potentially effective in the prevention of NPC metastasis and reduction of NPC radioresistance [6, 7]. However, the mechanism of LMP1-mediated radioresistance is not entirely clear.

Hypoxia-inducible factor 1 (HIF-1) regulates the response to radiotherapy treatment [8]. When HIF-1β is constitutively expressed, HIF-1α activity is increased not only by intratumoral hypoxia but also by genetic alterations under normoxic conditions [9]. One of the major targeted and activated genes is *vascular endothelial growth factor (VEGF)*, which plays a key role in tumor progression and angiogenesis. Moreover, VEGF has been reported to not only induce angiogenesis but also protects endothelial cells from the cytotoxic effects of irradiation and consequently increased tumor radioresistance [10]. Thus, HIF-1 plays a major role in the development of the tumor phenotype and affects tumor growth, angiogenesis, invasiveness, and metastasis [11]. Blockade of HIF-1 significantly increases tumor radiosensitivity by enhancing vascular destruction, thus demonstrating that HIF-1 plays a pivotal role in tumor radioresistance [8, 12].

Previous studies demonstrated that the p42/p44 signaling pathway is involved in LMP1-induced HIF-1 protein accumulation [13]. HIF-1 stimulates angiogenesis by up-regulating tumor cells to produce VEGF and other proangiogenic factors, which induces angiogenesis and protects the microvasculature from radiation-induced endothelial apoptosis [8, 14]. We previously reported that knocking down LMP1 could inhibit the expression and secretion of VEGF in NPC cells [15]. However, the molecular signaling mechanism(s) involved in LMP1-induced HIF-1/VEGF activation leading to radioresistance in NPC cells is not understood.

DNAzymes are single-stranded DNA catalysts that can be engineered to bind to their complementary sequence in the target messenger RNA (mRNA) and cleave the mRNA at predetermined phosphodiester linkages [16]. These catalysts have emerged as potential drug candidates because of their relative ease and low cost of synthesis, high stability, and flexible rational design features [17]. DZ1 is an LMP1-targeted DNAzyme that down-regulates the expression of LMP1 and inhibits signaling pathways that are abnormally activated by LMP1, thereby increasing apoptosis and inhibiting proliferation and increasing radiosensitivity of NPC cells, which are all substantially enhanced after treatment with a combination of DZ1 and irradiation [18]. Additional experiments showed that DZ1 decreased ataxia telangiectasia mutated (ATM) production by attenuating the binding of nuclear factor kappaB (NF-κB) to the *ATM* promoter and is involved in radioresistance [19]. Recent preclinical research studies have shown that DZ1 could down-regulate tumor vascular permeability [20].

Despite recent advances in understanding the molecular contribution of LMP1 to radiotherapy, important questions remain to be answered. For example, what are the distinct mechanisms involved in LMP1-induced VEGF expression? In this study, we determined whether LMP1-mediated radioresistance in NPC occurs through the HIF-1/VEGF pathway and elucidated the role of JNKs/c-Jun signaling involved in LMP1-induced HIF-1/VEGF activation in NPC cells.

## RESULTS

### EBV-LMP1 targets DNAzyme (DZ1) inhibits vasculature formation by down-regulating VEGF expression and secretion

**Figure 1:**
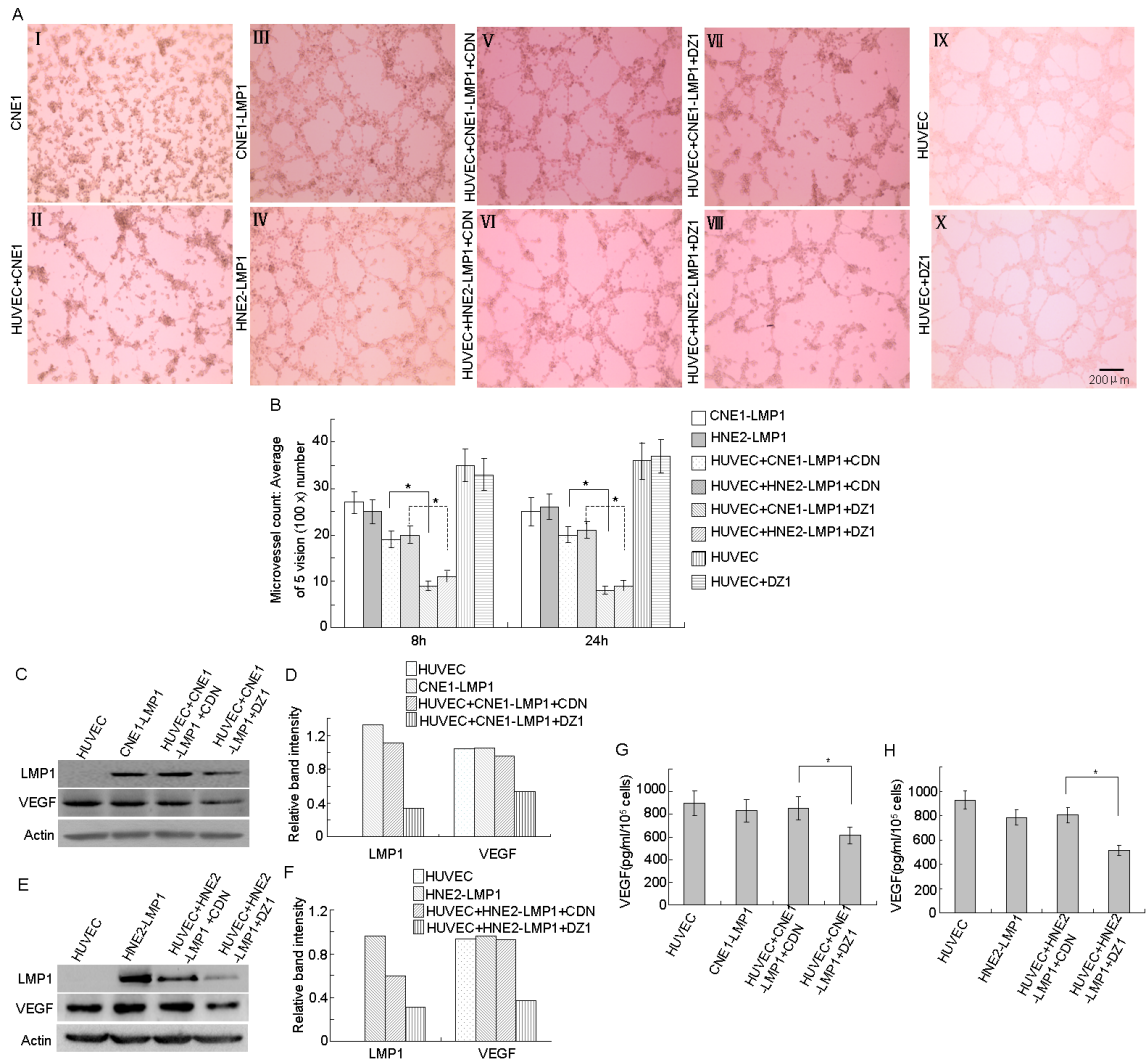
DZ1 inhibits vasculature formation by down-regulating VEGF expression and secretion CNE1-LMP1 or HNE2-LMP1 cells were transfected with DZ1 or CDN for 24 h. Then cells were trypsinized, collected and co-cultured with HUVECs (total number of cells = 1×10^5^) in a 24-well plate with 200 μl of embedded Matrigel and tube-formation was measured after 8 or 24 h. (A) Endothelial tube formation was assessed using light microscopy (8 h, magnification, 100×). (B) The average number of microtubules in 5 random horizons. (C, D, E, F) Western blotting analysis of LMP1 and VEGF protein expression in co-cultured cells and relative protein levels were quantified using ImageJ software. (G, H) The concentration of secreted VEGF in cells was measured using an ELISA kit. Data are expressed as means ± S.D. of three experiments and the asterisk (*) indicates a significant difference (*p* < 0.05) compared with the control.

Highly aggressive cancer cells can form patterned networks of matrix-rich tubular structures when cultured on three-dimensional (3-D) matrices *in vitro*, and these cancer cells together with endothelial cells form microtubules [21, 22]. To determine whether microtubule-forming ability is associated with LMP1 expression and VEGF activity, we performed a tube formation assay. The results showed that the well-differentiated LMP1-negative NPC cell line, CNE1, was not able to form tubular structures in Matrigel, whereas two LMP1-positive NPC cell lines, CNE1-LMP1 and HNE2-LMP1, could form tubular structures. Further, CNE1-LMP1 or HNE2-LMP1 cells and HUVEC endothelial cells could also be connected to each other to constitute a vascular network structure, suggesting that LMP1 might contribute to tumor vasculature formation in NPC (Fig. 1A, I-IV). Furthermore, the data indicated that co-cultures of CNE1-LMP1 or HNE2-LMP1 cells and HUVECs did not enhance the ability of cells to form tubular structures. This could be because HUVECs already express high levels of VEGF so that co-culturing HUVECs and CNE1-LMP1 cells are not able to induce higher levels of VEGF expression, resulting in a minimal effect on tube-formation. LMP1-positive NPC cells (1×10^5^), with down-regulated LMP1 expression, were co-cultured with HUVECs. After 8 or 24 h, the microtubule-forming ability of co-cultured cells was decreased by approximately one-half after treatment with DZ1 compared to control DNAzyme (CDN). This indicates that LMP1 knock-down by DZ1 in co-cultures resulted in reduced VEGF expression and inhibition of tube-formation (Fig. 1A, V-VIII, B). In addition, results showed that after treatment with DZ1, tube formation ability was not changed, which excluded the possibility that DZ1 might have an “off-target” effect on HUVECs (Fig. 1A, IX-X, B).

We then used Western blotting to examine the expression of LMP1 and VEGF proteins in co-cultured cells. The results showed that (Fig. 1C-F) treatment of LMP1-positive NPC cells with DZ1 reduced LMP1 expression compared to untreated control cells. The data also showed that co-culture of HUVECs and CNE1-LMP1/HNE2-LMP1 cells did not further increase VEGF expression. However, when LMP1 expression was reduced, co-culturing resulted in decreased VEGF expression, which indicates a requirement for LMP1 to maintain the expression level of VEGF in HUVECs. Compared with the control group, the secretion of VEGF into the medium of co-cultured cells was decreased after DZ1 treatment (Fig. 1G, H). These results indicated that DZ1 inhibits angiogenic activity by down-regulating VEGF secretion.

### The JNKs signaling pathway is involved in LMP1-induced HIF-1 and VEGF expression

**Figure 2:**
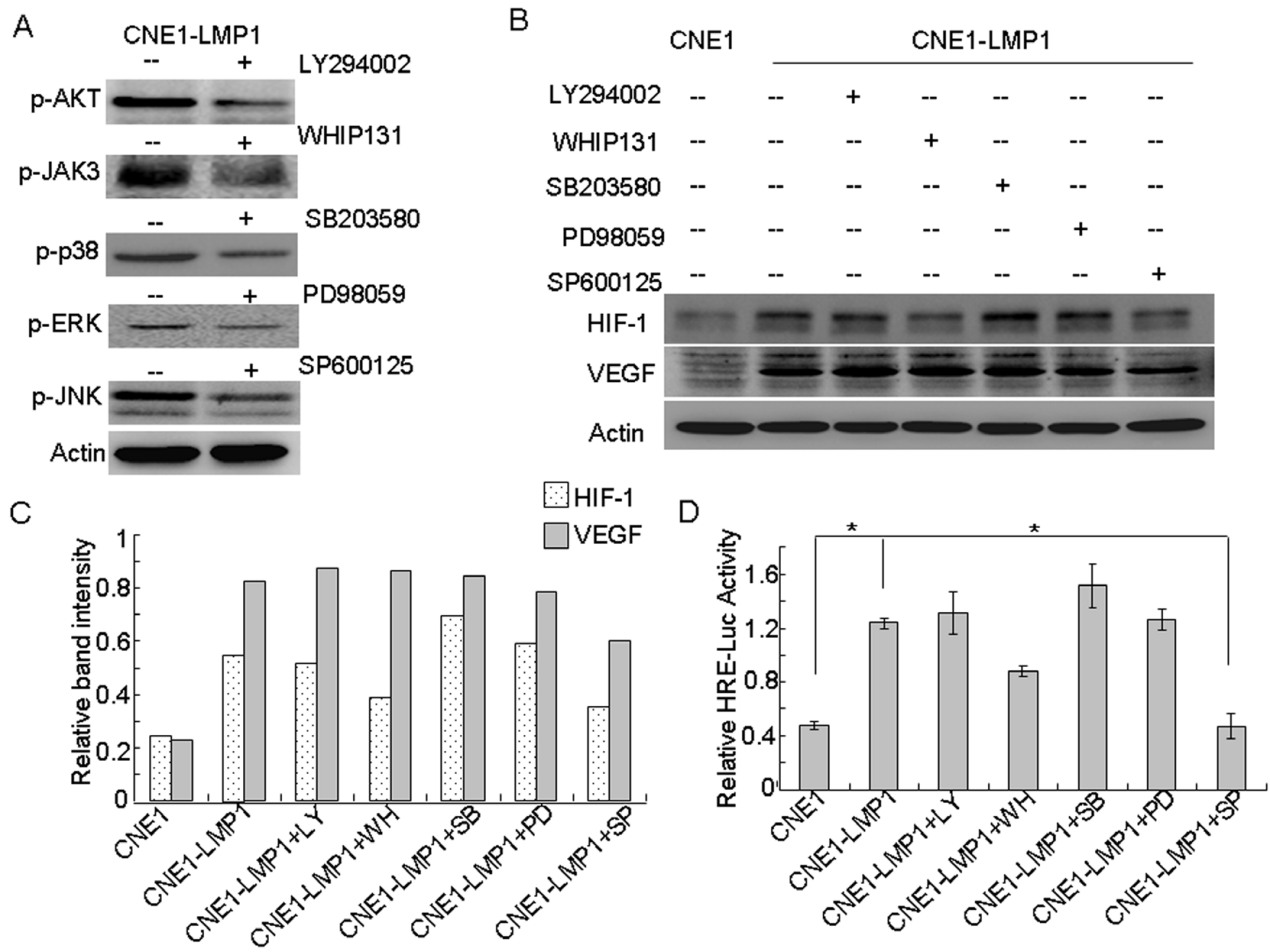
JNKs are involved in LMP1-induced HIF-1 and VEGF expression CNE1-LMP1 cells treated with the indicated concentrations of LY294002, WHIP131, SB203580, PD98059, or SP600125 and 400 μM CoCl_2_ for 24 h. (A) Western blotting was used to determine the effect of the inhibitors. (B) Western blotting were used to analyze the expression of HIF-1 and VEGF. β-Actin served as an internal control to confirm equal loading of proteins. (C) Relative protein levels were quantified using ImageJ software. (D) The *HRE* reporter gene was used to analyze HIF-1 transcriptional activity. Data are expressed as means ± S.D. of three experiments and the asterisk (*) indicates a significant (*p* < 0.05) difference compared with the control groups.

LMP1 activates various signaling cascades involved in proliferation, apoptosis and metastasis of NPC tumor cells [3]. To determine which pathway is mainly responsible for enhancing HIF-1/VEGF expression in NPC cells, we determined the expression of HIF-1 in CNE1-LMP1 cells treated with a variety of protein kinase inhibitors. The inhibitors included those acting against several protein kinases induced by LMP1, such as the PI3-K/AKT inhibitor (LY294002), a JAK3 inhibitor (WHIP131), a p38 inhibitor (SB203580), a MEK inhibitor (PD98059) and a JNKs inhibitor (SP600125) (Fig. 2A). The results showed that in CNE1-LMP1 cells, the expression of HIF-1 and VEGF increased and the expression of HIF-1 decreased after treatment with SP600125. In contrast, WHIP131 demonstrated a weak inhibitory activity, whereas the other compounds had very little effect. Importantly, the expression of VEGF decreased after treatment with SP600125, whereas the other inhibitors had no obvious effect (Fig. 2B, C). Furthermore, *HRE* reporter gene analysis showed that HIF-1 activity in CNE1 cells was significantly lower than that observed in CNE1-LMP1 cells (*p* < 0.05). In addition, pharmacological inhibition of JNKs by SP600125 resulted in significant (*p* < 0.05) decreases in HIF-1 activity. Although WHIP131 demonstrated a weak inhibitory activity, the other inhibitors had no effect (Fig. 2D). These results indicated that JNKs might be the main signaling pathway involved in the regulation of HIF-1/VEGF in NPC cells.

**Figure 3:**
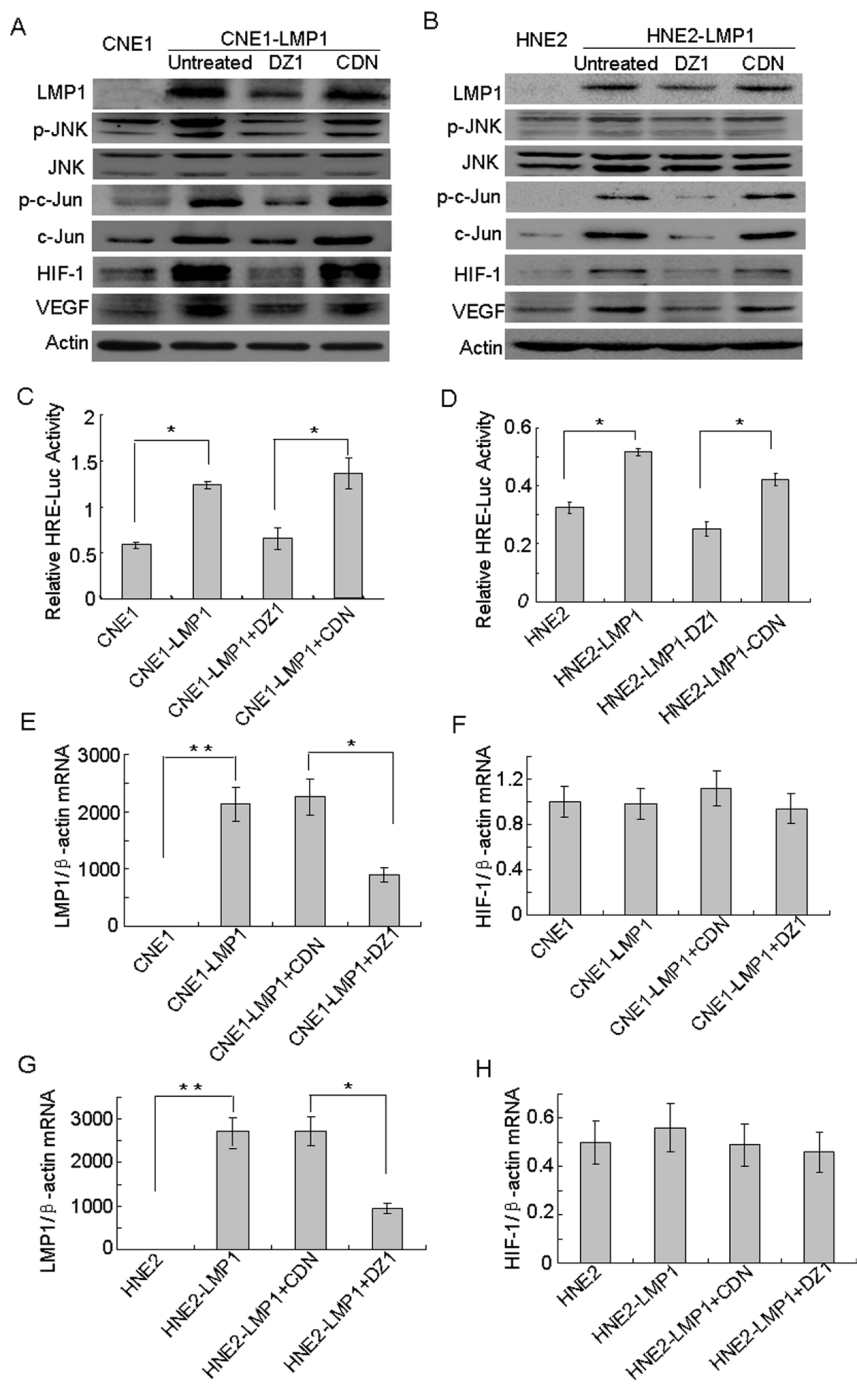
The JNKs/c-Jun signaling pathway is involved in LMP1-induced HIF-1 and VEGF expression Cells were treated with DZ1 or CDN and 400 μM CoCl_2_ for 24 h. (A,B) Western blotting was performed to analyze the expression of LMP1 and its downstream targets, including phosphorylated JNKs, JNKs, c-Jun, phosphorylated c-Jun, HIF-1 and VEGF in CNE1/CNE1-LMP1 cells and HNE2/HNE21-LMP1 cells. (C, D) HIF-1 activity levels were examined in these cells using the *HRE* reporter gene assay. Data were expressed as the means ± S.D. of three experiments. (E, F, G, H) Quantitative RT-PCR was used to analyze the mRNA expression of *LMP1* and *HIF-1* in CNE1/CNE1-LMP1 cells and HNE2/HNE2-LMP1 cells. The asterisk (* or **) indicates a significant (p<0.05 or p<0.01, respectively) compared with control groups.

To further confirm the direct involvement of JNKs/c-Jun signaling in the up-regulation of HIF-1 and VEGF expression mediated by LMP1, the two LMP1-positive NPC cell lines, CNE1-LMP1 and HNE2-LMP1, were treated with DZ1. Western blotting results showed that (Fig. 3A, B) phosphorylated JNKs (Thr183/Tyr185), total c-Jun and phosphorylated c-Jun (Ser73) were up-regulated in LMP1-positive NPC cells compared to LMP1-negative NPC cells. The total protein level of JNKs did not change. Moreover, expression of HIF-1 and VEGF was also up-regulated. In addition, results showed that after treatment with DZ1, phosphorylated JNKs, c-Jun and phosphorylated c-Jun were down-regulated, but the total protein level of JNKs was not obviously changed in LMP1-positive NPC cells compared to control cells. Furthermore, the expression of HIF-1 and VEGF was also down-regulated. In addition, *HRE* reporter gene analysis revealed that (Fig. 3C, D) HIF-1 activity in CNE1-LMP1 and HNE2-LMP1 cells was significantly higher than that observed in the corresponding LMP1-negative CNE1 and HNE2 cells (*p* < 0.05). Thus, HIF-1 activity in LMP1-positive NPC cells was decreased after treatment with DZ1 compared to control cells (*p* < 0.05). Taken together, these findings indicated that LMP1 could up-regulate the phosphorylation of JNKs and c-Jun, consequently promoting the expression of HIF-1 and further affecting the expression of VEGF in NPC cells.

To determine whether LMP1 could affect the expression of *HIF-1* mRNA, the two LMP1-positive NPC cell lines, CNE1-LMP1 and HNE2-LMP1, were treated with DZ1. The level of *HIF-1* mRNA was detected using quantitative PCR, but was not significantly changed in LMP1-positive NPC cells compared to LMP1-negative NPC cells (Fig. 3E-H). These results indicated that LMP1 did not affect the mRNA level of *HIF-1*. Thus, LMP1 increases the level of the HIF-1 protein not by increasing its transcription, but by amplifying its protein synthesis.

### LMP1 increases phosphorylation of JNKs and total HIF-1 and VEGF protein expression in NPC patient tissues

**Figure 4:**
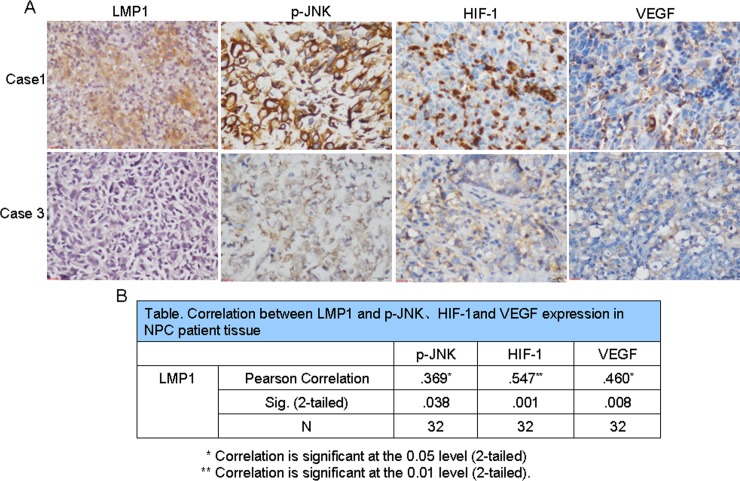
LMP1 up-regulates p-JNK, HIF-1 and VEGF expression in NPC patient tissues Immunohistochemical analysis was performed to examine the level of LMP1, phosphorylated JNKs, HIF-1 and VEGF expression in NPC patient tissues (magnification, 400×). (A) Tissue samples from Case 1 exhibited high expression of LMP1, phosphorylated JNKs, HIF-1 and VEGF, whereas tissue samples from Case 3 exhibited low levels of LMP1, phosphorylated JNKs, HIF-1 and VEGF. (B) Correlations between LMP1 and phosphorylated JNKs, HIF-1 and VEGF expression in NPC patient tissues.

Because of the role of LMP1 in regulating JNKs/HIF-1 signaling in NPC cells, we next determined whether LMP1-mediated phosphorylation of JNKs was associated with HIF-1/VEGF signaling in 32 clinical patient samples. Most of the samples presented different levels of LMP1 expression and the degree of expression of phosphorylated JNKs, HIF-1 and VEGF varied widely among cases (Fig. 4A). Furthermore, Spearman correlation analysis of the IHC data was performed and the relationship between LMP1 and phosphorylated JNKs, HIF-1, and VEGF was confirmed. The correlation coefficients for LMP1 and phosphorylated JNKs, HIF-1 or VEGF were 0.369, 0.547 and 0.460, respectively, and the p-values were 0.038, 0.001, and 0.008, respectively (Fig. 4B). These results indicated that a consistent positive relationship exists between LMP1 and phosphorylated JNKs, HIF-1 or VEGF expression.

### DZ1 and SP600125 inhibit the survival of LMP1-positive NPC cells after irradiation

**Figure 5:**
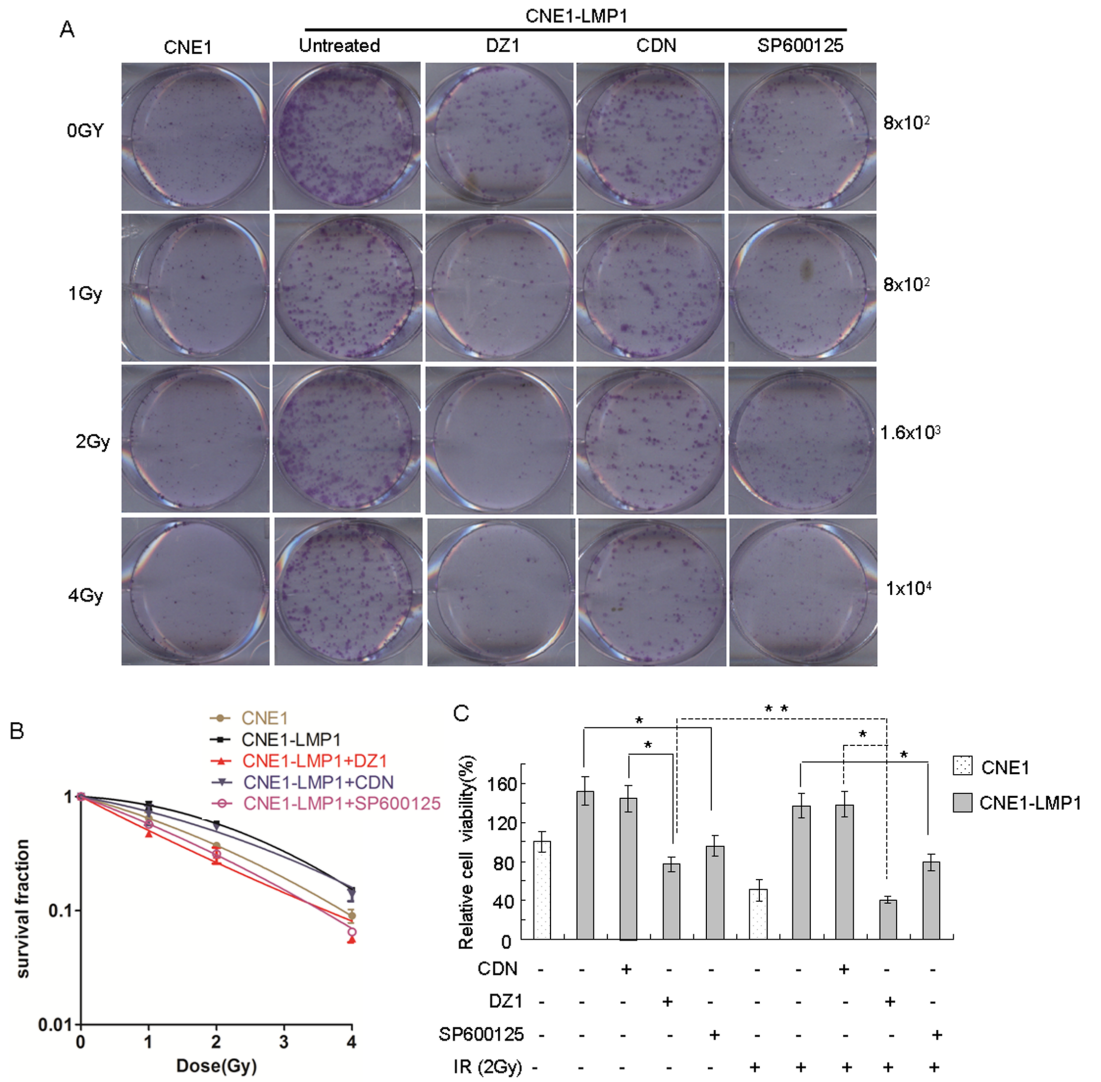
DZ1 and SP600125 inhibit the survival of LMP1-positive NPC cells after irradiation (A) Cells were treated with DZ1, CDN or SP600125 for 4 h and seeded at increasing numbers followed by irradiation at 0, 1, 2 or 4 Gy. The cells were incubated for 2 weeks before fixation, staining and colony quantification. Clonogenic assays were performed in triplicate. (B) Resulting survival curves were fit to the data using a linear quadratic model of radiation sensitivity. (C) The effect of DZ1, CDN or SP600125 on the sensitivity of NPC cells to radiation treatment was measured by MTS assay. CNE1 or CNE1-LMP1 cells were incubated with DZ1, CDN or SP600125 and then treated with radiation (2 Gy). The relative inhibition was calculated by comparing the OD value of each treatment group with the control group. Data are shown as mean values ± S.D. of three experiments. The asterisk (* or **) indicates a significant (*p* < 0.05 or *p* < 0.01, respectively) compared with control groups.

We next used the colony formation and MTS assays to determine whether suppressing the function of LMP1 and JNKs changes the survival of NPC cells with irradiation. The results showed that colony formation by LMP1-negative NPC cells, whether treated or not treated with irradiation, was weaker than that of LMP1-positive NPC cells, which indicated that LMP1 could increase cellular radioresistance. After treatment with DZ1 (2 μM) or SP600125 (20 nM), CNE1-LMP1 cells were rendered significantly more sensitive to radiation compared to untreated cells (Fig. 5A, B). Further, the results of an MTS assay indicated that radiation treatment could decrease the viability of all cell lines tested (Fig. 5C). Notably, the viability of CNE1-LMP1 cells treated with DZ1 or SP600125 was significantly lower compared with control cells. Furthermore, radiation treatment combined with DZ1 exerted a much stronger effect on viability of CNE1-LMP1 cells (*p* < 0.01). Overall, these findings indicated that inhibition of LMP1 or JNKs improved the tumor's response to radiation.

### Blockade of LMP1 with DZ1 suppresses radioresistance of NPCs

**Figure 6:**
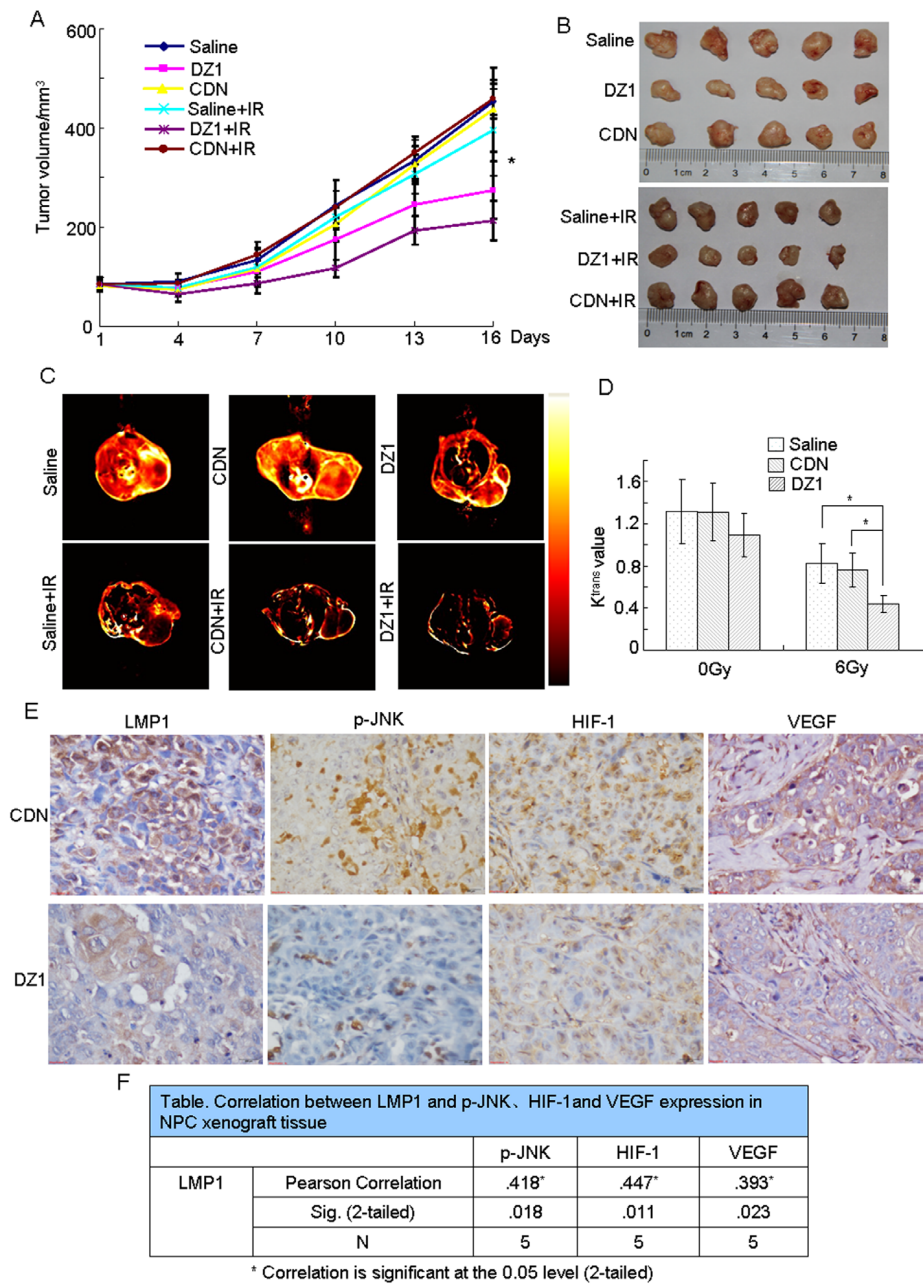
*In vivo* suppression of radioresistance of NPC by blockade of LMP1 with DZ1 The nasopharyngeal carcinoma xenograft model was established using CNE1-LMP1 cells. When the tumor volume reached 60-100 mm^3^, the animals were injected intra-tumorally twice a week (six times total) with 100 μg of DZ1 or CDN encapsulated by 3 μl of Fugene HD (20 μg per injection). The tumor volume was measured twice a week. (A) The tumor growth curve of each group in a nasopharyngeal carcinoma xenograft model. The asterisk (*) indicates the following: DZ1 vs. CDN (*p* < 0.05); DZ1+IR vs. CDN+IR (*p* < 0.01); DZ1 + IR vs. DZ1 (*p* < 0.05). (B) Tumor tissues were harvested at the end of the experiment. (C, D) MRI was conducted 3 days after DZ1 treatment. Representative images are presented. K^trans^ was generated from raw data of DCE-MRI using NordicICE software (version2.3.6). Values are expressed as mean values ± S.D. of triplicates and the asterisk (*) indicates a significant (*p* < 0.05) compared with the control. (E) Immunohistochemistry of DZ1- and CDN-treated tumors shows the expression of LMP1, phosphorylated JNKs, HIF-1 and VEGF (magnification, 400×). (F) Correlation between LMP1 and phosphorylated JNKs, HIF-1 or VEGF expression in NPC xenograft tissues.

To determine whether inhibition of LMP1 function by DZ1 effectively induces radiosensitivity *in vivo*, we treated athymic nude mice bearing CNE1-LMP1 xenografts with radiation, DZ1, or both. Treatments with DZ1 or radiation alone mildly decreased tumor growth, whereas combined treatment with radiation and DZ1 had an additive effect that suppressed tumor growth significantly more than that conferred by single treatments, (Fig. 6A, B).

We next used dynamic contrast enhanced magnetic resonance (DCE-MRI) to determine whether inhibition of LMP1 expression by DZ1 had any effect on tumor microvascular permeability (Fig. 6C). The parameter of K^trans^ derived from DCE-MRI has been widely used to evaluate anti-tumor drugs [23]. We compared the K^trans^ values of DZ1-, CDN- or saline-treated mice after radiotherapy and found significant differences between DZ1 and CDN (p = 0.028) or saline (p = 0.026) groups (Fig. 6D). However, significant differences between the DZ1 and control groups were not observed in non-irradiated animals. These results indicated that DZ1 inhibited LMP1 expression in mice, which might promote IR-mediated changes in tumor vasculature.

Furthermore, we also used immunohistochemistry to examine the effects of DZ1 treatment compared to CDN-treated controls. The data showed that LMP1 expression was substantially decreased in the CNE1-LMP1 xenograft tumors after DZ1 treated (Fig. 6E). Moreover, we found that treatment with DZ1 resulted in decreased expression of phosphorylated JNKs, HIF-1 and VEGF (Fig. 6E). The relationship between LMP1 and phosphorylated JNKs, HIF-1, and VEGF was confirmed. The correlation coefficients for LMP1 and phosphorylated JNKs, HIF-1 or VEGF were 0.418, 0.447 or 0.393, respectively, and the p-values were 0.018, 0.011, and 0.023, respectively (Fig. 6F). This inhibitory effect was consistent with the data obtained at the cellular level and from clinical patient samples.

## DISCUSSION

Radioresistance is a significant impediment in clinical settings for effective cancer therapy and is thought to be associated with multiple signaling pathways in different cancer types. Radiation increases hypoxia-inducible factor (HIF)-1 activity, which is a dominant governor of the tumor response to irradiation mediated by multiple mechanisms. These mechanisms include increased production of VEGF and other pro-angiogenic factors by tumor cells, which results in increased angiogenesis and protection of the microvasculature from radiation-induced endothelial apoptosis [24]. Associations between high levels of protein expression of HIF-1 in tumor tissues and a poor response to radiotherapy have been shown in multiple cancer types [25]. Moreover, HIF-1 activity is increased not only by intratumoral hypoxia but also by genetic alterations under normoxic conditions [9]. Herein, we showed that ectopic expression of LMP1 resulted in increased HIF-1/VEGF activity acting through the JNKs/c-Jun signaling pathway in CNE1-LMP1 cells rendering them more resistant to irradiation. Notably, knockdown of LMP1 by DZ1 in LMP1-positive NPC cells induced a more radiosensitive phenotype.

LMP1 induces HIF-1 protein expression and further amplifies VEGF expression, but this mechanism requires further elucidation. Wakisaka *et al.* demonstrated that LMP1 increases HIF-1 activity through the induction of HIF-1 protein synthesis without increasing its mRNA level, which is controlled by p42/p44 MAPK activity in a lymphoblastoid cell line [13]. In our system, LMP1-induced HIF-1 expression was not affected by PD98059, a pharmacological inhibitor of p42/p44 MAPK. These results indicated that the p42/p44 MAPK pathway is not involved in LMP1-induced HIF-1 activity. Several studies have reported functional interactions between increased JNKs activation and amplified HIF-1 activity in HUVECs and HepG2 cells [26, 27]. c-Jun reportedly cooperates with HIF-1 to activate the transcription of VEGF in a JNKs-phosphorylated fashion [26, 28], Thus, c-Jun could possibly modulate the HIF-1 protein level transcriptionally or post-transcriptionally. Herein the results showed that DZ1 blocked activity associated with phosphorylation of JNKs and suppressed both LMP1-induced total c-Jun and phosphorylated c-Jun expression, consequently decreasing HIF-1/VEGF expression. These results showed that JNKs/c-Jun signaling plays a central role in VEGF induction by LMP1. In addition to the level of transcription and protein synthesis of HIF-1, stabilization is an important consequence of post-translational modifications. HIF-1 is rapidly degraded by a von Hippel-Lindau tumor suppressor gene product (VHL)-mediated ubiquitin-proteasome pathway in normoxia conditions [29] and cobalt directly inhibits the prolylhydroxylase enzymes that direct HIF-1 to degradation [30]. In our experimental system, cobalt dichloride could suppress the degradation of the HIF-1 protein; thus, future work is needed to determine whether LMP1 specifically alters the stability of the HIF-1 protein.

In the present study, we provided evidence showing that LMP1 enhances protein levels of HIF-1 by amplifying its synthesis without increasing its mRNA level. Also, activation of the JNKs/c-Jun pathway is related to HIF-1 induction by LMP1 and LMP1 induces expression and secretion of VEGF, at least in part by activating HIF-1. These results are consistent with reports showing that JNKs, but not AKT, ERKs or p38 MAPK, are involved in the regulation of HIF-1 protein levels [31-33]. In some cases, however, AKT, ERKs, and p38 MAPK directly phosphorylate HIF-1 to regulate its transcriptional activity [34-36]. We believe that, depending on cell type, only some of these kinases are activated under specific experimental conditions.

Some aggressive tumor cells generate vasculogenic-like channels in the absence of endothelial cells or fibroblasts and the formation of the patterned microcirculation is termed vasculogenic mimicry (VM) [21, 22]. In addition, cancer cells together with endothelial cells form mosaic-like vessels. Furthermore, VM has been described in a variety of tumor types, including melanoma, breast, prostate, ovarian, and hepatocelluar carcinomas [37, 38]. Some studies have shown that patients with VM had a shorter 5-years survival rate and a higher blood-borne metastasis rate compared to non-VM patients [39, 40]. In our study, the data show that LMP1 could promote the ability of NPC cells to form vasculature. These results further illustrate the role of LMP1 in the tumorigenesis and angiogenesis of NPC.

DCE-MRI is a non-invasive imaging modality that can quantitatively evaluate tumor vascularity and tumor vascular parameters. DCE-MRI has been developed as a powerful tool for tumor characterization and to assess the early efficacy of anticancer therapies, including anti-angiogenesis and radiation therapies [41]. This method monitors the pharmacokinetic uptake and wash-out of a MRI contrast agent within the extracellular space of tumor tissues, which can be used to evaluate a vascular permeability coefficient (K^trans^, volume transfer constant between the blood plasma and the EES). This has been shown to correlate with tumor perfusion and angiogenesis. Preliminary reports have also shown that K^trans^ provides an early measure of response to therapy, with responding tumors showing a reduction in K^trans^ values [42]. Furthermore, by measuring the *in vivo* tumor vessel permeability with DCE-MRI, DZ1 was shown to efficiently reduce K^trans^, which indicated that suppression of LMP1 in NPCs could change tumor vasculature, resulting in an inhibition of tumor growth and increased radiosensitivity.

Clear advantages of DNAzyme compared to other RNA-targeting agents include its ease of chemical synthesis, good stability and high catalytic turnover [43]. Recently, a clinical trial demonstrated the anti-tumor and radiosensitizing effects of DZ1 [17]. Here, we demonstrated HIF-1/VEGF activation induced by LMP1 in NPC cells occurs through JNKs /c-Jun-dependent pathways, which resulted in decreased angiogenesis and increased radiosensitivity in NPC cells.

**Figure 7:**
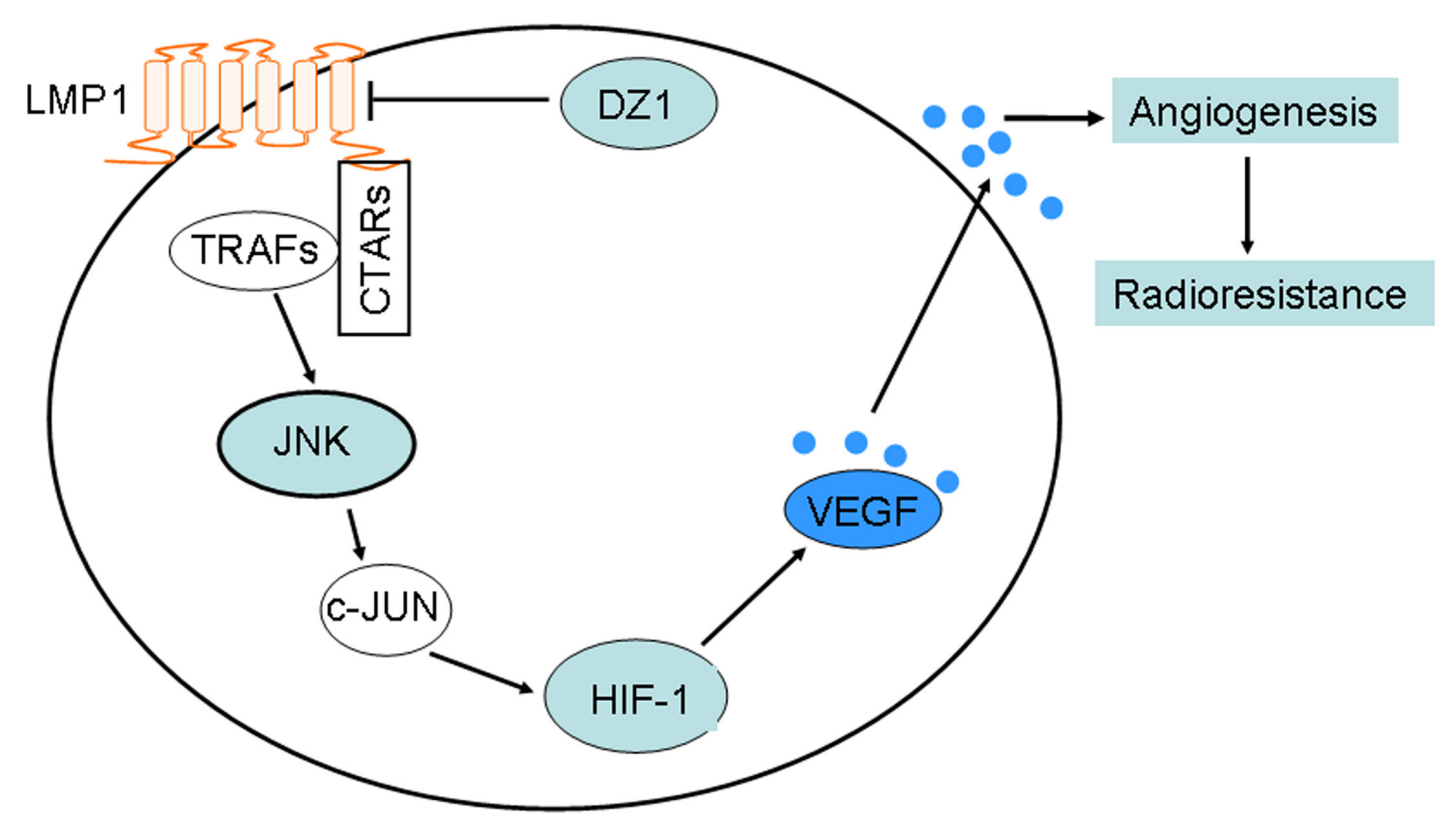
A schematic illustrating the potential DZ1 therapeutic pathways The C-terminal activation regions (CTARs) in the cytoplasmic C-terminus of LMP1, which directly bind TNF receptor-associated factors (TRAFs), constitutively engage the JNKs signaling pathways. JNKs induces synthesis of the HIF-1 protein through its downstream target, c-Jun. Further, HIF-1 activates the expression and secretion of vascular endothelial growth factor (VEGF) by the specific binding of HIF-1 to the hypoxia-responsive element (*HRE*). Then, VEGF increases angiogenesis and radioresistance. Knocking down expression of LMP1 with DZ1 will inhibit angiogenesis and enhance radiosensitivity through this pathway in NPC.

## METHODS

### Cell culture and DNAzyme transfection

Human umbilical vein endothelial cells (HUVEC, ATCC CRL-1730) were purchased from Xiangya Central Experiment Laboratory. CNE1 is an LMP1-negative NPC cell line and CNE1-LMP1 is a stable LMP1-integrated cell line [44]. HNE2 is an LMP1-negative NPC cell line, and HNE2-LMP1 is a cell line that constitutively expresses LMP1 after the introduction of full-length LMP1 cDNA [45]. Cells were grown in RPMI 1640 (Gibco BRL, Gaithersburg, MD) supplemented with 10% fetal bovine serum (FBS). DZ1 and control DNAzyme (CDN), which was designed based on DZ1 by introducing two mutations in the catalytic core, were synthesized by Oligos Etc. Inc. (Wilsonville, OR) and transfected as previously described [19].

### Tube formation assay

A matrigel tube formation assay was developed for testing of the anti-angiogenic activity of DZ1 based on the differentiation of HUVECs on Matrigel (BD Biosciences, Franklin Lake, NJ). Briefly, we first transfected CNE1-LMP1 or HNE2-LMP1 cells with DZ1 or CDN, and 24 h later, cells were trypsinized and centrifuged at 600 × g for 5 min. Approximately 5×10^4^cells and the same number of HUVECs (total number of cells was 1×10^5^) were seeded in each well of a 24-well plate with 200 μl of embedded Matrigel. Next, the cells were incubated for 8 or 24 h, and the extent of microtubule-forming ability was examined using low magnification microscopy.

### Western blotting analysis

Proteins were extracted from cells in RIPA buffer (50 mM Tris-HCl pH 8, 150 mM NaCl, 0.1% SDS, 1% NP-40, 0.5% sodium deoxycholate, 0.57 mM PMSF and 1 μg/ml aprotinin). Protein samples (50 μg) were separated by 12% SDS-PAGE, transferred onto nylon membranes and immunoblotted with primary antibodies. Binding of primary antibodies was detected using peroxidase conjugated secondary antibodies (Santa Cruz, CA) and developed with an enhanced chemiluminescence detection kit (Pierce ECL, Thermo Scientific, Pittsburgh, PA.). The study employed antibodies against LMP1 (M0897, DAKO, Carpinteria, CA), c-Jun (sc-1694, Santa Cruz, CA), phosphorylated c-Jun (Ser 73, sc-7981, Santa Cruz), JNKs (9252, Cell Signaling, Beverly, MA), phosphorylated JNKs (Thr183/Tyr185, 9251, Cell Signaling), HIF-1(sc-53546, Santa Cruz), VEGF (sc-152, Santa Cruz) and β-actin (sc-8432, Santa Cruz). The relative protein levels were quantified using ImageJ software (NIH).

### Measurement of vascular endothelial growth factor (VEGF)

Cell culture medium was collected, and the concentration of VEGF was measured using an ELISA kit (Quantikine, R&D Systems, Minneapolis, MN), according to the manufacturer's instructions.

### HIF-1 activity analysis

Cells were transfected with the *HIF-1-luciferase* reporter plasmid (SABiosciences, QIAGEN, Valencia, CA) using Lipofectamine 2000 (Invitrogen, Carlsbad, CA). After incubation for 24 h, cells were transfected with DZ1 or CDN and were subsequently incubated with cobalt (400 μm/L) for 24 h. Luciferase activity (Dual-Luciferase Reporter Assay, Promega, Madison, WI) was measured using an AutoLumat LB 9505c luminometer (Berthold Analytical Instruments, Nashua, Germany).

### Quantitative RT-PCR

Total RNA purification and the reverse transcription process have been previously described [46]. Quantitative PCR was performed with iTaq^TM^ SYBR Green Supermix with ROX (172-5850, Bio-Rad) using an ABI 7500 instrument. The primers used to detect HIF-1 were: 5′-CGTTCCTTCGATCAGTTGTC-3′ (forward) and 5′-TCAGTGGTGGCAGTGGTAGT-3′ (reverse); LMP1 were: 5′-CGTTATGAGTGACTGGACTGGA-3′ (forward) and 5′-TGAACAGCACAATTCCAAGG-3′ (reverse). Primers for detecting β-actin were 5′-CATGTACGTTGCTATCCAGGC-3′ (forward) and 5′-CTCCTTAATG TCACGCACGAT-3′ (reverse).

### Immunohistochemistry

Immunohistochemistry (IHC) was performed using a Histomouse SP Broad Spectrum DAB kit (Invitrogen–Zymed, Carlsbad, CA). Paraffin sections were immunostained using a streptavidin peroxidase procedure after microwave antigen retrieval. The signal was detected using a diaminobenzidine solution. The stained sections were independently examined by two of the authors (L.L. and D.F.). A semi-quantitative evaluation of the positivity of each protein by IHC was performed using a method described as follows [47]: the percentage of positive cells was divided into five grades (percentage scores): ≤10% (0), 11–25% (1), 26–50% (2), 51–75%(3), and >75% (4). The intensity of staining was divided into four grades (intensity scores): no staining (0), light brown (1), brown (2), and dark brown (3). Staining positivity was determined by the formula: overall scores = percentage score × intensity score.

### Clonogenic Assay

A clonogenic assay was performed as previously described [19]. The data were analyzed using the linear-quadratic model. The surviving fraction was calculated as the ratio of the plating efficiency of the treated cells compared to control cells.

### Cell viability assay

Cells were cultured in 96-well plates. Cell viability was examined using the CellTiter 96 Aqueous One Solution (MTS) Reagent (Promega, Fitchburg, WI) according to the manufacturer's instructions.

### Mouse xenograft model

All mice were maintained and manipulated according to strict guidelines established by the Medical Research Animal Ethics Committee, Central South University, China. Six-week-old female athymic nude mice (BALB/C) were injected with 5×10^6^ CNE1-LMP1 cells. Tumor volumes were calculated using the formula: (length*width*height)*(π/6). When the tumor volume reached 60–100 mm^3^, the animals were injected intra-tumorally with 100 μg of DZ1 or CDN with 3 μl of Fugene6 (Roche, Indianapolis, IN) or saline only once every three days. In groups of mice subjected to irradiation treatment (IR), 6 Gy local irradiation was administered at 24 h after the first DZ1 injections.

### DCE-MRI Imaging

MRI was performed 3 days after DZ1 treatment. All data were acquired on superconducting magnetic resonance imaging scanners (Signa HDx 3.0T, GE Healthcare, Milwaukee, WI) equipped with a coil that was 2.0 inches in diameter for animal experiments. Mice were anesthetized using 10% chloral hydrate. DCE-MRI scanning was performed as previously described [23]. The raw data of DCE-MRI were analyzed using NordicICE software (version2.3.6, NordicNeuroLab, Bergen, Norway).

### Statistical analysis

All statistical analyses were performed using SPSS16.0. The data shown are the mean values of at least three different experiments and are expressed as the mean ± S.D. (standard deviation). The Student's t-test was used for comparison and *p* < 0.05 was considered statistically significant.
